# Sudden Cardiac Death in Athletes: Facts and Fallacies

**DOI:** 10.3390/jcdd10020068

**Published:** 2023-02-05

**Authors:** Jennie Han, Andrea Lalario, Enzo Merro, Gianfranco Sinagra, Sanjay Sharma, Michael Papadakis, Gherardo Finocchiaro

**Affiliations:** 1 Department of Cardiology, Royal Brompton Hospital, London SW3 6NP, UK; 2 Department of Cardiology, Azienda Sanitaria Universitaria Integrata Giuliano Isontina (ASUGI), University of Trieste, 34123 Trieste, Italy; 3 Cardiovascular Sciences Research Centre, St. George’s, University of London, London SW17 0RE, UK

**Keywords:** cardiomyopathy, sudden cardiac death, sudden arrhythmic death syndrome, channelopathy, athlete’s heart, sports cardiology

## Abstract

The benefits of exercise for cardiovascular and general health are many. However, sudden cardiac death (SCD) may occur in apparently healthy athletes who perform at the highest levels. A diverse spectrum of diseases is implicated in SCD in athletes, and while atherosclerotic coronary artery disease predominates in individuals of >35 years of age, primary cardiomyopathies and ion channelopathies are prevalent in young individuals. Prevention of SCD in athletes relies on the implementation of health policies aimed at the early identification of arrhythmogenic diseases (such as cardiac screening) and successful resuscitation (such as widespread utilization of automatic external defibrillators and training members of the public on cardiopulmonary resuscitation). This review will focus on the epidemiology and aetiologies of SCD in athletes, and examine fallacies in the approach to this controversial field. Furthermore, potential strategies to prevent these tragic events will be discussed, analysing current practice, gaps in knowledge and future directions.

## 1. Introduction

The beneficial effects of regular physical activity on general health are well established. Several studies have demonstrated a lower all-cause mortality and incidence of cardiovascular diseases, cancer and metabolic conditions in individuals who engage in regular exercise [1,2,3,4,5,6,7,8,9,10]. Despite these premises, sudden cardiac death (SCD) may occur in apparently healthy individuals, including athletes. Sudden cardiac death is defined as an unexpected death from cardiac causes that occurs within one hour (or within 24 h in unwitnessed cases) from the onset of an acute change in cardiovascular status in the absence of external causal factors [11]. In Western countries, SCD is estimated to account for up to 13–20% of all deaths [11,12].

The epidemiology and aetiology of SCD in athletes is a controversial area. In this review, we will discuss the epidemiological burden of SCD in athletes and the most common underlying aetiologies. We will examine landmark studies and historical fallacies as well as review policies aimed at preventing these tragic events.

## 2. Methods

The authors approached this topic with the following research questions: (1) What are the incidences, determinants and causes of sudden cardiac death or aborted sudden death in athletes? (2) What are the controversial issues on this topic? (3) What are the issues in differential diagnosis between “athlete’s heart and cardiac conditions that predispose to sudden cardiac death and what are the possible means to prevent sudden death in athletes? Therefore, a systematic search through the web-based engine PubMed was conducted to identify all studies meeting the eligibility criteria. Most relevant studies answering the main research questions were selected. Finally, results are presented systematically, taking in account the complexity of the topic.

## 3. Incidence of Sudden Cardiac Death in Athletes

Several studies have reported on the epidemiology of SCD in athletes, describing an extremely variable incidence (Table 1) [13,14,15]. Differences among studies are probably due to many factors and most importantly the methodologies used. A unifying definition of “athlete” does not exist, and this inevitably results in a selection bias and heterogeneous approach. The methods of recording SCD events are many (some studies rely on media reports or insurance claims, others on national or regional registries) and differ among studies, as do the approaches to the post-mortem investigations aimed at clarifying the cause of death. Furthermore, some studies include only SCDs, while others also include sudden cardiac arrests (SCAs) in the calculation of events. Finally, public health measures, such as pre-participation cardiac screening and the public availability of automatic external defibrillators (AEDs), are different among countries, and these factors may lead to variability in the assessed incidence of events [16].

### 3.1. Relationship with Age

The incidence of sports-related SCD increases with age in both non-competitive and competitive sports. The last decades have witnessed an increasing number of “older” (age > 35 years) athletes participating in organized competitive sport events. In the United States, in 2013, 54% of the 19,025,000 participants in competitive running races were ≥35 years of age [26]. As reported by Risgaard et al. [27], the estimated incidence of SCD was 0.47–1.21 per 100,000 person years in young (age ≤ 35 years) competitive athletes compared to 6.64 per 100,000 person years in older (age > 35 years) athletes [27,28]. The incidence of SCD in Minnesota high school athletes (age 12 to 19 years) over 19 academic years was lower: 0.24 per 100,000 athlete years [24]. Harmon et al. [20] described an annual incidence of SCD of 1:43,770 in athletes who were a part of the United States National Collegiate Athletic Association (NCAA) (age 17–23 years). Marijon et al. [29] reported on patients from 35 to 65 years of age who had experienced SCA during sport activities. The mean age was 51.1 ± 8.8 years and sports-related sudden cardiac arrests (SrSCA) occurred in a small proportion of SCA cases (5%), more frequently in middle-age individuals and those with at least one cardiovascular risk factor and a known heart disease.

### 3.2. Relationship with Sex

Several studies consistently showed that SCD is more common in male compared with female athletes (Table 1). A study by Corrado et al. [18] on more than 110,000 athletes reported an incidence rate of SCD of 2.6/100,000 person years in male athletes compared to 1.1/100,000 person years in female athletes. On the other side of the Atlantic, Maron et al. [21] showed that out of 1049 SCDs in young competitive athletes (median age 19 ± 6 years) in the United States, only 11% occurred in females. This trend also persists despite the increase in female participation in different sports, among ethnicities and at any age [18,21].

A large regional registry in the United Kingdom found that among 748 cases of SCD in individuals who engaged in sport activities (>3 h of organized physical training per week), only 98 (13%) were women [30]. The same study demonstrated a significant lower incidence of death during intense exertion in female athletes compared to male athletes (58% versus 83%; *p* < 0.001) [30].

The hypotheses explaining the higher incidence of SCD in male athletes include the effect of sex on physiological cardiac adaptation to exercise and chamber remodelling; the higher male prevalence of myocardial fibrosis, which may constitute a substrate for life-threatening arrhythmias [30,31,32]; hormonal factors (where oestrogens may be protective in females); the higher male prevalence of atherosclerotic coronary artery disease; and psychological factors, particularly considering the tendency of males to reach levels of exhaustion and engage in addictive behaviours more frequently [31,33,34].

### 3.3. Relationship with Ethnicity

Recent studies have shown an association between ethnicity and SCD (Table 1). The NCAA database reported an overall SCD rate among athletes of 1:43,770 student athletes per year [24]. The incidence rose to 1:17,696 athletes per year among black college athletes and to 1:5284 athletes per year in the college basketball Division I African American players subgroup [24]. It was estimated that young African American athletes had a threefold increase in the rate of SCD compared to white athletes, with an incidence rate of 5.6/100,000 per year. These epidemiological trends are consistent with other studies on young athletes in the United States [22,35,36].

A recent prospective study conducted through the National Center for Catastrophic Sports Injury Research in the United States reported on 331 confirmed cases of SCA and SCD (158 survivors, 173 fatalities) and showed that the annual incidence rate among African American male NCAA Division I basketball players was 1:2087 athlete years, the highest compared to other ethnic groups [23].

### 3.4. Relationship with Type and Intensity of Sport

The type and the level of effort may impact the risk of developing life-threating arrhythmias if an underlying cardiac condition is present. It appears that strenuous exercise performed by competitive athletes, as opposed to recreational exercise, has the potential to lead to a higher risk of SCD (Table 1) [17,21,25]. Sollazzo et al. [12] showed a rate of 1:100,000 SCDs among competitive athletes versus 0.32:100,000 in those practicing leisure activities or recreational sports. Toresdahl et al. [25] reported a relative risk of 3.6 of sudden cardiac arrest in high school campus student athletes compared to their sedentary counterparts. In contrast, a study from Maron et al. [37] reported a threefold higher incidence of SCD among young individuals who did not engage in intense exercise compared to competitive athletes. Sudden cardiac deaths were 8-fold more common in non-athletes [37].

In the United States, the highest incidence of SCD has been reported in basketball players [18,19,21]. In contrast, in Europe, football leads to most sports-related SCD events. According to a study from Malhotra et al. [38], the incidence of SCD was 6.8 per 100,000 athlete years among young football players who were screened with an ECG and health questionnaire at the age of 16. An Italian study reported that 45% of all SCD events in athletes occurred whilst playing football, with a similar percentage (39%) reported in Israel [39,40]. This may be reflective of the greater popularity of both amateur and professional soccer in Europe compared to the United States, which may have influenced the size of the sporting population.

## 4. Causes of Sudden Cardiac Death

**Figure 1:**
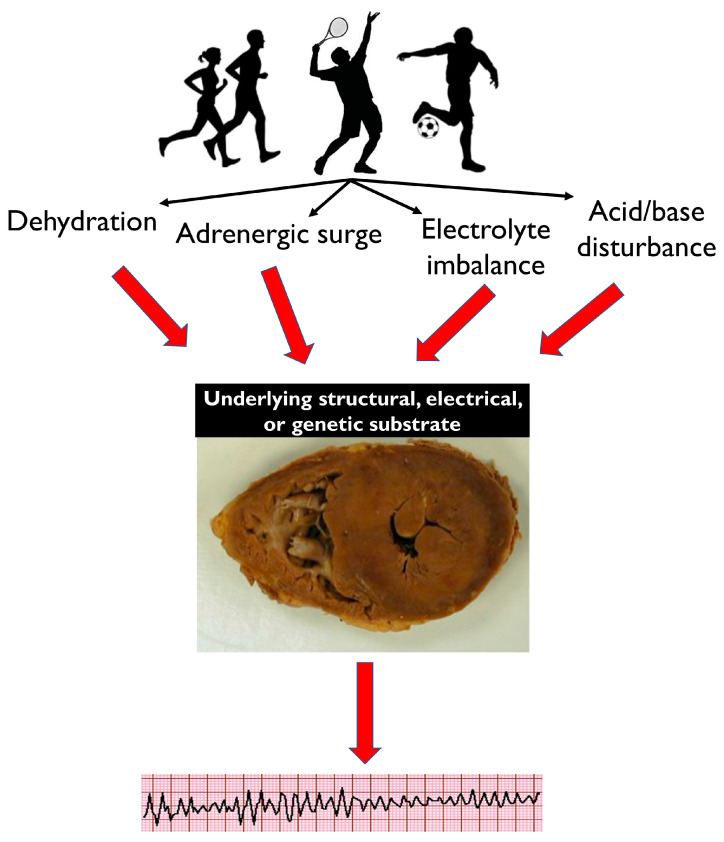
Dehydration, adrenergic surge, electrolyte imbalance and acid/base disturbance are common physiological effects of intense exercise. While these are well tolerated by healthy athletes, they may cause potentially fatal arrhythmias in individuals with an underlying electrical, structural or genetic pathological cardiac substrate. This image of a heart at post-mortem is suggestive of HCM, and this is used as an example.

**Figure 2:**
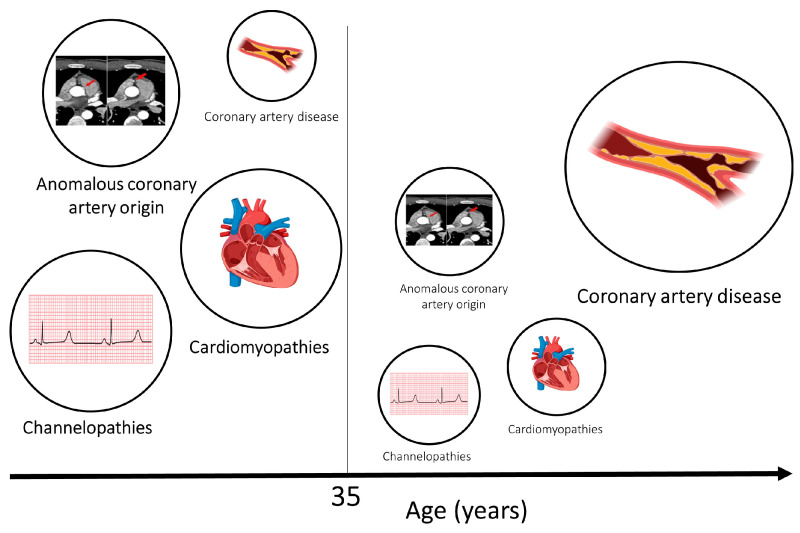
Aetiologies of SCD in athletes and age. Cardiomyopathies, channelopathies and congenital anomalies of the coronary arteries are prevalent causes in younger individuals. Atherosclerotic coronary artery disease is the most common cause of sudden cardiac death and sudden cardiac arrest in older individuals. The size of the circles relate to the relative frequency of SCD caused by the respective pathology. Age 35 has been used as a threshold as this is the most frequently used age in the literature; however, a clear line in terms of age is difficult to draw.

Common physiological effects of intense exercise, such as dehydration, adrenergic surge, electrolyte imbalance and acid/base disturbance, may not be well tolerated by athletes with a pathological electrical or structural substrate, resulting in potentially fatal arrhythmias (Figure 1). A diverse spectrum of diseases is implicated in SCD, with variable prevalence dependent on the demographics of the victims and the circumstances of death. The majority of SCDs are attributable to atherosclerotic coronary artery disease and generally manifest in individuals in the fourth decade onward (Figure 2). The primary cardiomyopathies and ion channelopathies are the predominant causes of SCD in the young (<35 years). The inherited nature of these conditions underscores the need for cardiac evaluation of first-degree relatives of the deceased. Post-mortem examination is an essential first diagnostic step to guiding clinical evaluation of surviving relatives toward inherited structural diseases or primary arrhythmogenic syndromes. Often, the post-mortem assessment is not performed by expert cardiac pathologists through standardized protocols, and this may result in inaccuracy in establishing the cause of death [41,42,43].

**Figure 3:**
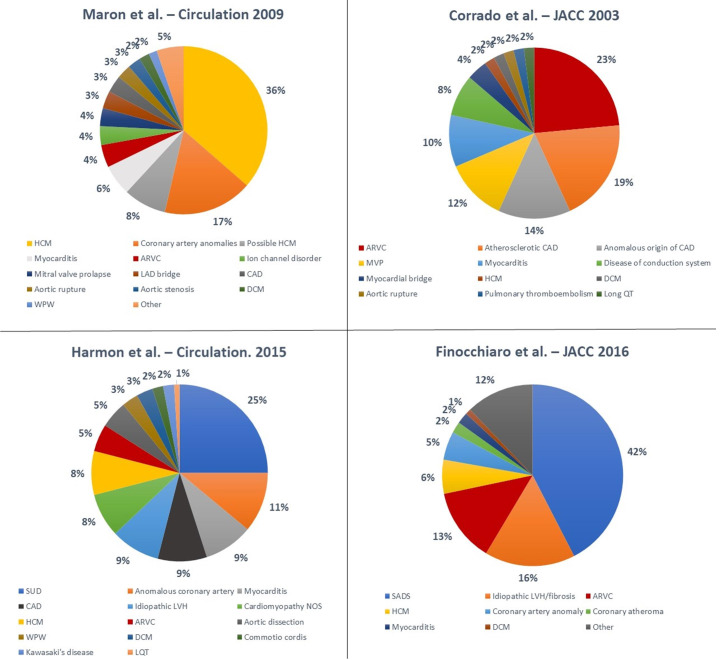
Summary of main studies describing the causes of SCD in athletes. Abbreviations: ARVC—arrhythmogenic right ventricular cardiomyopathy; CAD—coronary artery disease; DCM—dilated cardiomyopathy; HCM—hypertrophic cardiomyopathy; LAD—left anterior descending artery; LQT—long QT; LVH—left ventricular hypertrophy; MVP—mitral valve prolapse; NOS—not otherwise specified; SADS—sudden arrhythmic death syndrome; SUD—sudden unexplained death; WPW—Wolff–Parkinson–White [18,21,41,45].

Numerous studies have been conducted to elucidate the underlying aetiologies of SCD in athletes (Figure 3) [18,21,41,44,45]. The variability in terms of results is significant. Hypertrophic cardiomyopathy (HCM) is traditionally considered the most common cause of SCD in young athletes in the United States; Maron et al. [21] reported on the National Registry of SCD in Athletes (based in the United States) and showed that HCM accounted for 36% of all SCD events in young athletes. These data are based on a large cohort but are limited by the fact that an autopsy was not performed in all athletes and was carried out by an expert cardiac pathologist only in the minority of cases.

Recent studies have reported different results (Table 2). A study by Corrado et al. [18] showed that in the Veneto region in Italy, arrhythmogenic right ventricular cardiomyopathy (ARVC) was the most common cause of SCD in young athletes (23% of cases), while HCM accounted for only 2% of deaths. Mandatory pre-participation screening (using the ECG) in Italy may explain the reported low incidence of HCM. In fact, ECG abnormalities are common in HCM, and the disqualification of affected athletes may have prevented SCDs [46].

Eckart et al. [44] examined 902 cases of SCD in active military personnel from the Department of Defense in the United States. These individuals were in active service, and therefore were required to maintain a certain level of fitness. In young individuals (<35 years), the heart appeared structurally normal at the post-mortem examination in 41% of cases. Hypertrophic cardiomyopathy accounted for only 13% of cases. In older individuals the most common cause of death was atherosclerotic disease (73%). This study was retrospective, and most of autopsies were conducted by a local medical examiner with no regular involvement of a specialist cardiac pathologist. Harmon et al. [45] reported that a structurally normal heart at the post-mortem examination was the most common finding (25%) in 64 cases of college athletes who died suddenly. Coronary artery anomalies were the second most frequent cause (11%), and HCM accounted for 8% of the cases. 

Finocchiaro et al. [41] described a cohort of 357 athletes who died suddenly in the United Kingdom where the post-mortem examination was performed by an expert cardiac pathologist. The most common finding at the post-mortem examination was a normal heart (42% of cases), followed by myocardial disease including idiopathic left ventricular hypertrophy (LVH) and idiopathic fibrosis (16%), ARVC (13%), and HCM (6%). Coronary artery anomalies were found in 5% of cases.

The interpretation of the post-mortem results is a complex task and uncertainty may exist about the exact significance of certain pathological findings and their causal relationship with SCD. The clinical significance of a structurally normal heart with normal toxicology (defined as sudden arrhythmic death syndrome (SADS)) in the context of SCD is not fully understood. In SADS cases, death is most likely caused by primary arrhythmia syndromes, such as long QT syndrome, Brugada syndrome or catecholaminergic polymorphic tachycardia. Genetic testing of the deceased proband (molecular autopsy) and family screening of family members may help in providing a unifying diagnosis. Recent studies showed that up to 50% of families of SADS victims are affected by an inherited cardiac condition (usually a channelopathy) that can be linked with the SCD in the proband [47,48,49]. In some cases of SADS or unexplained cardiac arrest, pathogenic variants in cardiomyopathy-related genes are found, which raises the possibility of an arrhythmic phenotype preceding a fully expressed structural phenotype [50].

Idiopathic fibrosis and idiopathic LVH are common autopsy findings in young athletes who have died suddenly. The clinical significance of these entities is unclear as they may be incidental and innocent bystanders or may constitute substrates for potentially fatal arrhythmias. Finocchiaro et al. [51] investigated whether idiopathic LVH and familial HCM are part of the same disease. These authors comprehensively assessed first-degree family members of 46 decedents with idiopathic LVH and found that none fulfilled diagnostic criteria for HCM, suggesting that idiopathic LVH is a distinct disease entity.

Although genetic and inherited cardiac conditions are the predominant causes of SCD in young individuals, the contribution of non-genetic and “acquired” factors may be relevant. Specifically, drugs, alcohol and smoking may act as second hits in predisposed individuals, resulting in maladaptation and potentially fatal arrhythmias. The use of performance-enhancing drugs has progressively increased recently. These drugs are constantly under review by the World Anti-Doping Agency (WADA) [52]. Side effects are many and depend on the type of substance, the amount and the duration of use, leading in some cases to tragic consequences, including SCD [53].

### Circumstances of Death

Sudden cardiac death in athletes often occurs during exercise, but it can also occur at rest and sometimes during sleep. A recent study on athletes in the United Kingdom showed that 61% of athletes died suddenly during exertion, including a small proportion of individuals (4%) who died during altercation. Of the individuals who died at rest, one-third died while sleeping [41]. Certain cardiac conditions, such as arrhythmogenic cardiomyopathy (AC) and coronary artery anomalies, often lead to SCD during exertion [41]. Intense exercise has been shown to be particularly deleterious in AC, where the higher risk of fatal arrhythmias is often accompanied by a worsening of the phenotype [54,55]. Coronary artery anomalies comprise many anatomical subtypes. A retrospective analysis of 30 cases with an anomalous origin of the coronary artery revealed that anomalous left coronary artery arising from the right sinus of Valsalva was mostly associated with SCD during exercise (73% of SCDs occurred during exercise, compared to 18% in the anomalous right coronary artery arising from the left sinus of Valsalva) [56]. In contrast, in cases of SADS, SCD occurs more often at rest or during sleep [57].

## 5. Prevention of SCD in Athletes

Sudden cardiac death in athletes may be prevented through the implementation of policies aimed at identifying cardiac conditions that may pose a risk in asymptomatic individuals (screening) and policies that increase the likelihood of successful resuscitation of cardiac arrests.

### 5.1. Pre-Participation Cardiac Screening

Both the American Heart Association/American College of Cardiology (AHA/ACC) and the European Society of Cardiology (ESC) recommend pre-participation cardiac screening with the aim of identifying cardiac conditions that pose a risk of SCD [58,59,60,61,62]. There is no global consensus on whether or how cardiac screening should be performed, with high variability among countries, sports governing bodies and level of competition. Both American and European guidelines suggest that medical history and physical examination should be part of the cardiac screening assessment [57,58,59,60,61]. However, while the 12-lead ECG is the key investigation proposed by the ESC, this test is not recommended by the AHA, which instead focuses on personal and family history and physical examination [58,59,60]. Corrado et al. [16] showed that the implementation of a mandatory pre-participation cardiac screening program with the use of the ECG led to a significant decrease in the incidence of SCD in athletes. In the Veneto region of Italy, the annual incidence of SCD in athletes decreased by 89% after cardiac screening become compulsory in 1982. In contrast, Maron et al. [63] analysed 13 cases of SCD that occurred in high school student athletes in Minnesota over a 26-year period and reported that only 4 (31%) individuals had cardiovascular conditions that could have been reliably detected through cardiac screening with history, examination and ECG. Furthermore, Steinvil et al. [64] showed that the effect of implementing mandatory pre-participation cardiac screening with ECG and exercise testing in Israel in 1997 did not lead to a significant change in documented events of SCD in competitive athletes. This study relied on the systematic search of two main newspapers in Israel, which constitutes a significant limitation as data were not collected through a prospective database, resulting in a possible underestimation of the events.

If initial tests are suspicious for cardiac disease, further investigations, such as echocardiogram, cardiovascular magnetic resonance, cardio-pulmonary exercise testing and family testing, are recommended. In the presence of a suspicious phenotype, genetic testing may be helpful for diagnosis and risk stratification [58]. Although specific genetic testing can be helpful, in the absence of a suspicious phenotype or a positive proband, genetic screening is not routinely carried out [65]. This is because it has a low yield and variants of unknown significance, which may not be clinically significant, may be found [65].

While pre-participation screening may be ethically justified to prevent SCD in athletes, there are a number of complex ethical issues relating to disqualification decisions [66]. Athletes, especially those competing at very high levels, may perceive disqualification based on the results generated by screening as discrimination on the basis of a medical condition. Moreover, disqualification can cause significant psychological stress in athletes, which may affect their well-being in the long term.

A shared decision-making process regarding sport participation is always advisable, taking into account the lack of robust evidence on the risk of exercise in individuals with cardiac disease. There are certain conditions and situations wherein exercise appears to be deleterious and carries significant risk. These include AC prior cardiac arrest or unexplained syncope, symptomatic/obstructive HCM, DCM with significant impairment of systolic function and/or high-risk genotypes (lamin A/C—filamin C) [58].

The use of mass cardiac screening in athletes remains controversial. The most cited issues are those relating to the economic sustainability of a nationwide screening program, the uncertain benefits in terms of SCD reduction, the potential for false positives and consequential unnecessary disqualifications and the fact that screening does not prevent all cardiac deaths among young athletes [38,61].

### 5.2. Differential Diagnosis between “Athlete’s Heart” and Pathological Cardiac Conditions

**Figure 4:**
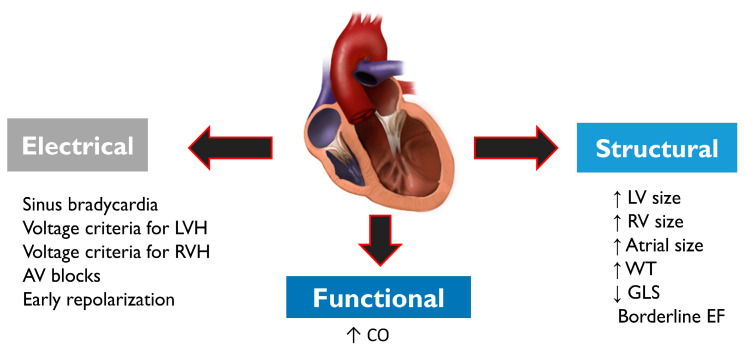
Structural, electrical and functional physiological changes commonly observed in athletes. Physiological changes may overlap with cardiac conditions that may pose a risk of SCD, and therefore differential diagnosis is very important. Abbreviations: AV—atrioventricular; CO—cardiac output; EF—ejection fraction; GLS—global longitudinal strain; LV—left ventricle; LVH—left ventricular hypertrophy; RV—right ventricle; RVH—right ventricular hypertrophy; WT: wall thickness.

Pre-participation cardiac screening in athletes may lead to further investigations aimed at ruling out cardiac disease. Diagnosis is often complex as athletes usually exhibit a series of electrical, structural and functional physiological changes (Figure 4), which may overlap with cardiac pathology. Structural changes on echocardiogram include an increased cardiac chamber size and myocardial wall thickness. Electrical changes on ECG include sinus bradycardia or arrhythmia, first-degree or Mobitz type 1 atrioventricular block, voltage criteria for ventricular hypertrophy, incomplete right bundle branch block, T-wave inversion and J-point elevation with ascending ST segments [67]. Functional changes include increased diastolic filling and stroke volume. Demographic factors such as sex and ethnicity may influence cardiac adaptation to exercise in athletes; concentric remodelling and hypertrophy of the left ventricle are more prevalent in males, while females more frequently exhibit eccentric LV hypertrophy [32].

### 5.3. Role of cardiopulmonary resuscitation (CPR) and AEDs

A cornerstone in the prevention of SCD in athletes is the immediate availability of quality CPR performed by bystanders and AEDs. A study based on a dedicated Luxembourg nationwide database showed that the ratio of survival among patients receiving bystander CPR during a cardiac arrest was about 50% [17]. In contrast, all cases of cardiac arrests not having CPR were fatal. The greatest determinant of survival after SCA is the time from collapse to defibrillation, with survival rates declining from 7% to 10% per minute for every minute lost [19].

AEDs should be promptly available so that a first shock can be applied within 3 minutes of the collapse [68]. The role of education in the general population is fundamental, as well as the deployment of external defibrillators in public areas and training and sports grounds to exponentially increase the chance of better outcomes [20]. A recent investigation by Karam et al. [69] described a substantial stability of incidence of sport-related SCA between 2005 and 2018; in the first 2-year period of the study, the estimated incidence was 7.00 per million inhabitants/years compared to 6.24 per million inhabitants/years in the last 2 years. The increased education of the general population in terms of bystander cardiopulmonary resuscitation (CPR) and use of public automated external defibrillators (AEDs) led to a significant improvement in survival to hospital discharge rates (23.8% in the first period compared to 66.7% in the last) [69].

## 6. Conclusions

Sudden cardiac death is a tragic event that may affect apparently healthy individuals, including athletes. Underlying cardiac conditions may pose a risk of SCD, and the combination of an abnormal electrical or structural substrate with the physiological changes and demands associated with intense exercise may result in fatal arrhythmias. The incidence of SCD is higher in males and in older athletes. While primary cardiomyopathies and channelopathies are the main causes of SCD in young athletes, atherosclerotic coronary artery disease is the prevalent cause of fatal events in veteran athletes. The underlying aetiologies of SCD vary among cohorts, emphasizing the complexity of autopsy interpretation in this context. A structurally normal heart is increasingly found at post-mortem examination in young athletes who die suddenly, suggesting that primary arrhythmia syndromes may often play a role.

Pre-participation cardiac screening is a possible means to identify cardiac conditions that may cause SCD in athletes. While European guidelines recommend the use of a 12-lead ECG, American guidelines focus on personal and family history and physical examination. Cardiac screening in athletes remains controversial, with critics raising concerns regarding the economic sustainability, the cost-effectiveness and the ethical issues related to disqualification. Although early identification of silent cardiac diseases may help in preventing these tragedies, the implementation of policies aimed at the widespread use of AEDs and early CPR remain crucial.

## Figures and Tables

**Table 1:** Main studies reporting on Incidence of SCD in athletes.

Authors—Year—Country Study Design	Case Identification	Denominator	SCD or SCA + SCD	Years Studied (n° of Years)	Population	Incidence	Age Range (Mean Age)	Number of Deaths
**Besenius—2022—Luxembourg** **Prospective** [17]	**Declaration by the general population (online questionnaires, public media, informed patients followed by the national cardiology institute and cardiac re-education centres, direct witnesses, and other publicly available information)**	**Cases of SrSCD on national territory or outside Luxembourg suffered by a Luxembourgish resident or sports license holder**	**SCD + SCA** **(+ myocardial infarctions and acute coronary syndromes)**	**2015–2019** **(5)**	**Physically active population (competitive and non-competitive)**	**2.6 cases/year/100,000 physically active inhabitants**	**17–80** **(49.7)**	**43 (17 fatal)**
Corrado—2003—Italy Prospective [18]	Mandatory death reporting	Registered Italian athletes	SCD	1979–1999 (20)	Athletes and young people	1:47,600 athlete 1:142,900 young people	12–35 (23)	55
Drezner—2005—USA Retrospective	Survey answered by 244/326 Div. I NCAA institutions	Number of athletes at surveyed schools	SCD		College athletes	1:67,000		5
Drezner—2009—USA Cross-sectional survey [19]	1710 high schools with AEDs surveyed for SCA or SCD	Number of student athletes reported by schools	SCA + SCD	2006–2007 (within 6 months of survey)	High school athletes	1:23,000 SCA + SCD 1:46,000 SCD	14–17 (16)	14
Drezner—2014—USA Retrospective	Media reports	NFHS	SCA + SCD	2003–2013 (10)	High school athletes	1:153,846 SCD 1:71,428 SCA 1:21,277 male basketball	14–18	6 SCD 7 SCA
Harmon—2011—USA Retrospective [20]	Parent Heat Watch database, NCAA Resolutions list, insurance claims	Participation data from NCAA	SCD	2004–2008 (4)	College athletes	1:43,770	18–26 (20)	37
Harmon—2014—USA Retrospective	Media reports	NFHS	SCA + SCD	2007–2013 (5)	High school athletes	1:63,988 SCA 1:41,662 male 1:33,815 male basketball	14–18	74 SCD 35 SCA
**Holst—2010—Denmark Retrospective**	**Review of death certificates, then autopsies if available**	**Denmark population statistics**	**SCD**	**2000–2006** **(7)**	**Athletes and young people**	**1:82,645 SrSCD** **1:26,595 general population**	**12–35** **(26)**	**15 SrSCD** **470 SCD**
Maron—1996—USA Retrospective	US Registry for Sudden Death in Athletes	“Unavoidable selection bias and certainly significantly underestimated”	SCD	1985–1995 (10)	Athletes		12–40 (17)	134
Maron—1998—USA Retrospective	Insurance claims	Minnesota State High School League	SCD	1985–1997 (12)	High school athlete	1:217,000 overall 1:129,000 male 0 for female	16–17 (16.5)	3
Maron—2003—USA Retrospective	US Registry for Sudden Death in Athletes	Not possible b/c of selection bias	SCD	1985–2000 (25)	Athletes		9–40 (17)	286
Maron—2009—USA Retrospective [21]	US Registry for Sudden Death in Athletes	Not described (estimated 10.7 million athletes < 39 participating in sports during 2000-2006)	SCA + SCD	1980–2006 (27)	Athletes	1:163,934	8–39 (18)	690
Maron—2013—USA Retrospective	US Registry of Sudden Death in Athletes	Minnesota State High School League	SCD	1986–2011 (26)	High school athletes	1:150,000	12–18 (16)	13
Maron—2014—USA Retrospective [22]	US Registry for Sudden Death in Athletes and NCAA resolutions list for cardiac cases	Participation data from NCAA	SCD	2002–2011 (9)	College athletes	1:62,000 presumed 1:83,000 confirmed	17–26 (20)	64
Peterson—2021—USA Prospective [23]	National Center for Catastrophic Sports Injury Research in collaboration with national sports organizations	Participation data from National Federation of State High School Associations and NCAA	SCD + SCA	2014–2018 (4)	Competitive athletes	High school level: 1:65,872 athlete years NCAA level: 1:50,768 athlete-year	11–29 (16.7)	331 SCA + SCD 173 SCD
Roberts—2013—USA Retrospective [24]	MSHSL records	Participation data from MSHSL records	SCD	1993–2012 (19)	High school athletes	0.24 per 100,000 athlete years	12–19 (“most aged 15 to 18”)	4
Steinvil—2011—Israel Retrospective	Retrospective review (2 Israeli newspapers by 2 media researchers)	45,000 registered competitive athletes in 2009, extrapolating the growth of the Israeli population for those aged 10–40 born after 1985 based on a figure allowed for presumed doubling of the sport-participating population	SCD	1985–2009 (24)	Athletes	1st 1:39,370 2nd 1:37,593	12–44 (24)	24
Toresdahl—2014—USA Prospective [25]	2149 schools with cases of SCA + SCD occurred on school campus	Number of student athletes reported by schools	SCA + SCD	2009–2011 (2)	High school athletes	1:87,719 SCA + SCD 1:57,000 male SCA + SCD	14–18	18 SCA + SCD 2 SCD
Van Camp—1996—USA Retrospective	National Center for Catastrophic Injury Research and media database	17 sports, participants in NCAA, NFHS, NAIA, NJCAA, added together, conversion factor (1.9 for high school and 1.2 for college) to account for multisport athletes, used “based on discussions with representatives from the national organisations”	SCD	1983–1993 (10)	College athletes and high school	1:300,000	17–24 (17)	100

**In bold:** studies focused on SrSCD or SCA during sports activities. ADHD—attention-deficit hyperactivity disorder; AED—automatic external defibrillators; CV—cardiovascular; DOD—Department of Defense; EMS—emergency medical services; MSHSL—Minnesota High School League; NAIA—National Association of Intercollegiate Athletics; NCAA—US National Collegiate Athletic Association; NFHS—National Federation of State High School Associations; NJCAA—National Junior College Athletic Association; SCA—sudden cardiac arrest; SCD—sudden cardiac death; SrSCD—sports related sudden cardiac death.

**Table 2:** Causes of death in athletes.

Author	Year	Country	Study Design	Case Identification	SCD or SCA + SCD	Years Studied	Population of SCD	Cause of Death	Post-Mortem Assessment
Maron [21]	2009	USA	Retrospective cohort	US National Registry of Sudden Death in Athletes	SCA + SCD	1980–2006	690 competitive athletes	HCM (36%), anomalous origin of coronary artery (17%), possible HCM (8%), myocarditis (6%), ARVC (4%), channelopathies (4%)	Autopsy reports by local medical examiners—some primary pathological material selectively analysed
Corrado [18]	2003	Italy	Prospective cohort	Center for Sports Medicine in Padua	SCD	1979–1999	51 competitive athletes	ARVC (23%), coronary atherosclerosis (19%), anomalous origin of coronary artery (14%), MVP (12%), myocarditis (10%), conduction disease (8%), HCM (2%).	Post-mortem assessment of heart by expert cardiac pathologist
Eckart [44]	2011	USA	Retrospective cohort	Department of Defense Cardiovascular Death Registry	SCD	1998–2008	902 uniformed personnel from the Department of Defense	<35y—SUD (41.3%), atherosclerotic disease (23.2%), HCM (12.8%), myocarditis (5.7%), DCM (4.7%) ≥35y—atherosclerotic disease (73.2%), SUD (10.6), DCM (3.5%), HCM (3.1%)	Autopsy by medical examiners and Department of Defense, clinical cause of death adjudicated by authors
Harmon [45]	2015	USA	Retrospective cohort	National Collegiate Athletic Association database (Parent Heart Watch and insurance claims)	SCD	2003–2013	64 NCAA (college) Athletes	SUD (25%), coronary artery abnormalities (11%), myocarditis (9%), coronary artery disease (9%) DCM (8%), HCM (8%), idiopathic LVH/possible HCM (8%), ARVC (5%)	Autopsy reports reviewed by panel of experts including cardiac pathologist
Finocchiaro [41]	2016	UK	Retrospective cohort	Cardiac Risk in the Young database	SCD	1994–2014	357 individuals engaging in regular sport activity (>3 h training/week)	Normal post-mortem (42%), LVH/fibrosis (16%), ARVC (13%), HCM (6%), coronary artery anomaly (5%)	Post-mortem assessment of heart by expert cardiac pathologist

ARVC—arrhythmogenic right ventricular cardiomyopathy; DCM—dilated cardiomyopathy; HCM—hypertrophic cardiomyopathy; LVH—left ventricular cardiomyopathy; NCAA—National Collegiate Athletic Association; SCA—sudden cardiac arrest; SCD—sudden cardiac death; SUD—sudden unexplained death.

## Data Availability

Not applicable.
